# Different Approaches for Treating Multilevel Cervical Spondylotic Myelopathy: A Retrospective Study of 153 Cases from a Single Spinal Center

**DOI:** 10.1371/journal.pone.0140031

**Published:** 2015-10-13

**Authors:** Xiumao Li, Liang Jiang, Zhongjun Liu, Xiaoguang Liu, Hua Zhang, Hua Zhou, Feng Wei, Miao Yu, Fengliang Wu

**Affiliations:** 1 Orthopaedic Department, Peking University Third Hospital, HaiDian District, Beijing, 100191, China; 2 Research Center of Clinical Epidemiology, Peking University Third Hospital, HaiDian District, Beijing, 100191, China; UCLA, UNITED STATES

## Abstract

**Objective:**

The optimal surgical treatment for multilevel cervical spondylotic myelopathy (MCSM) remains controversial. This study compared the outcomes of three surgical approaches for MSCM treatment, focusing on the efficacy and safety of a combined approach.

**Methods:**

This retrospective study included 153 consecutive MCSM patients (100 men, 53 women; mean age ± standard deviation, 55.7 ± 9.4 years) undergoing operations involving ≥3 intervertebral segments. The patients were divided into three groups according to surgical approach: anterior (n = 19), posterior (n = 76), and combined (n = 58). We assessed demographic variables, perioperative parameters, and clinical outcomes ≥12 months after surgery (20.5 ± 7.6 months), including Japanese Orthopaedic Association (JOA) score, improvement, recovery rate, and complications.

**Results:**

The anterior group had the most favorable preoperative conditions, including the highest preoperative JOA score (12.95 ± 1.86, *p* = 0.046). In contrast, the combined group had the highest occupancy ratio (48.0% ± 11.6%, *p* = 0.002). All groups showed significant neurological improvement at final follow-ups, with JOA recovery rates of 59.7%, 54.6%, and 68.9% in the anterior, posterior, and combined groups, respectively (*p* = 0.163). After multivariable adjustments, the groups did not have significantly different clinical outcomes (postoperative JOA score, *p* = 0.424; improvement, *p* = 0.424; recovery rate, *p* = 0.080). Further, subgroup analyses of patients with occupancy ratios ≥50% showed similar functional outcomes following the posterior and combined approaches. Overall complication rates did not differ significantly among the three approaches (*p* = 0.600). Occupancy ratios did not have a significant negative influence on postoperative recovery following the posterior approach.

**Conclusions:**

If applied appropriately, all three approaches are effective for treating MCSM. All three approaches had equivalent neurological outcomes, even in subgroups with high occupancy ratios. Further investigations of surgical approaches to MCSM are needed, particularly prospective multicenter studies with long-term follow-up.

## Introduction

Cervical spondylotic myelopathy (CSM) is a common degenerative disease that is characterized by compressions in the cervical region of the spine and results in progressive neural cell loss and neurological deterioration in around 20% to 60% of patients [1]. Generally, 1- or 2-level spondylosis and/or severe cervical kyphosis are treated using an anterior surgical approach, whereas multilevel stenosis and dorsal pathology are treated using a posterior surgical approach [2, 3]. Although several studies have described different surgical strategies for CSM, the optimal surgical choice for multilevel cervical spondylotic myelopathy (MCSM, 3 or more intervertebral segments involved) remains undetermined [4].

The combined posterior-anterior surgical approach can be used to achieve complete decompression and restoration of sagittal alignment in one stage, and represents an alternative option for treating MCSM with large ventral compressions [5, 6]. Previous studies of the anterior, posterior, and combined surgical approaches have suggested that the combined approach may offer the best neurological outcomes without significantly increasing complications [7]. The combined approach’s major disadvantages include its technical complexity, prolonged surgical times, and increased blood loss [8]. Some surgeons prefer using a combined approach for MCSM, while others insist that a posterior approach is sufficient for the majority of cases. Abumi [9] and Riew [10] suggest performing surgery with a posterior approach, waiting for 2 or 3 weeks, and then performing surgery with an anterior approach, if needed.

In light of the current uncertainty in the literature, we conducted a retrospective study to compare the neurological outcomes associated with anterior, posterior, and combined surgical approaches in the treatment of MCSM. Our study focused most heavily on the efficacy and safety of the combined approach. Furthermore, we examined the relationship between the canal occupancy ratio and patients’ neurological recovery, in order to validate the indication for a combined surgical approach in patients with high occupancy ratios.

## Materials and Methods

### Ethics Statement

This study was approved by the Ethical Committee of the Third Hospital of Peking University and conducted according to the principles of the Declaration of Helsinki. All of the participants provided signed, informed consent at admission, before their data were stored in the hospital database and used for research purposes.

### Patient Population

We retrospectively reviewed the medical records of all 676 patients who underwent cervical decompressions that were performed by the spinal surgery team at our hospital between January 2012 and March 2014. A preliminary cohort of 163 consecutive MCSM patients were included in the present study, based on the following criteria: (1) clinically and radiographically confirmed CSM, with or without ossification of the posterior longitudinal ligament (OPLL); and (2) at least 3 intervertebral segments included in the operation [11, 12]. The exclusion criteria were as follows: (1) spine tumor or trauma, (2) moderate or severe cervical kyphotic deformity (C2-7 Cobb angle > 10°), (3) concomitant symptomatic disorders of the thoracic or lumbar spine, and (4) a history of previous cervical spine surgery [13]. The minimum follow-up period was 12 months.

### Surgical Management

The choice of surgical approach depended on both the characteristics of the cord compression and the experiences of the surgeons. Generally, the anterior approach was used for compressions caused by only 3 degenerative discs and/or osteophytes. The posterior approach was employed in cases of congenital and/or degenerative cervical stenosis involving 3 or more levels, with or without ligamentum flavum hypertrophy. Finally, the combined approach was used to treat multilevel cervical spondylosis accompanying severe ventral compression. We arbitrarily defined severe ventral compression as a canal occupancy ratio of 50% or greater, or between 40% and 50% in combination with slight cervical kyphosis [14]. All operations were performed by the same surgical team. The anterior approach consisted of anterior cervical discectomy and fusion (ACDF) and anterior cervical corpectomy and fusion (ACCF) techniques, while the posterior approach used open-door laminoplasty [15]. The one-stage combined posterior-anterior approach was performed under general anesthesia in the prone position initially (posterior stage), followed by the supine position (anterior stage). Postoperatively, each patient remained in bed until his or her drainage tube was removed. In the 4 subsequent weeks, the patients were allowed to walk with a soft cervical collar for comfort.

### Clinical assessments

The patients’ neurological outcomes were assessed using the Japanese Orthopaedic Association (JOA) scoring system [16], and the recovery rate was calculated according to the Hirabayashi method: recovery rate (%) = (postoperative JOA score–preoperative JOA score)/(17 [full score]–preoperative JOA score) × 100% [17]. Additionally, a set of treatment-related complications was documented, including dysphagia, cerebrospinal fluid (CSF) leakage, C5 palsy, and wound complications [18]. When evaluating postoperative complications, axial symptoms were considered to be present if a patient complained about any kind of discomfort in the postoperative period, including pain, fatigue, stiffness, and tightness in the neck, the shoulders, or the periscapular region [15]. In contrast, axial pain specifically referred to the experience of nuchal or periscapular pain. Dysphonia, nerve root paresthesia, and other complications were defined as transient if they were only observed during the perioperative period (within 30 days after surgery) [19].

### Radiological assessments

Preoperative radiological assessments included radiography, computed tomography (CT), and magnetic resonance imaging (MRI) examinations of the cervical spine. Cervical alignment was measured in the profile of neutral lateral plain radiographs using Ishihara’s curvature index (CI) [20]. The degree of cervical stenosis was indicated by the mean Pavlov ratio at levels C3 through C6, as described previously [21]. The anteroposterior (AP) diameter [22] of the spinal cord and the occupancy ratio [23] of the spinal canal at the site of maximal compression were measured using T2-weighted magnetic resonance images (T2WI). The appearance of increased signal intensity (ISI) of the spinal cord on T2WI was confirmed by the same surgical team [22].

### Statistical analysis

Data were statistically analyzed using SAS version 9.1.3 (SAS Institute Inc., Cary, NC). Results for categorical and continuous variables are presented as counts with percentages and means ± standard deviations, respectively. The chi-square (χ^2^) test, analysis of variance, the t-test, or non-parametric analyses were used to assess differences among approaches, as appropriate [7]. Spearman’s rank correlation test was used to assess the association between 2 variables [24]. To account for key differences in baseline predictive variables, adjusted multivariable analyses were performed using multiple linear regression [13]. Unless stated otherwise, a 2-tailed *p* of less than 0.05 was considered statistically significant.

## Results

Of the 163 preliminary subjects, 6 patients were excluded because of missing preoperative MRI data, 2 patients died at around 1 year after surgery due to unrelated causes, and another 2 patients were lost to follow-up. Therefore, at the final follow-up visit (mean, 20.5 ± 7.6 months; range, 12–36 months), neurological function assessments were obtained in 153 (98.7%) of the 155 eligible subjects, including 19 (12.4%), 76 (49.7%), and 58 (37.9%) patients treated with anterior, posterior, and combined approaches, respectively. The follow-up periods in these three groups were 18.0 ± 6.6 months, 20.3 ± 8.0 months, and 21.6 ± 7.6 months, respectively (*p* = 0.201, Table 1). A 2-year follow-up was obtained for 3 patients (15.8%) in the anterior group, 28 patients (36.8%) in the posterior group, and 26 patients (44.8%) in the combined group (*p* = 0.075, Table 1). No significant differences were found among the three groups in terms of demographic parameters, such as gender ratios, ages, or durations of symptoms (Table 1).

**Table 1:** Demographic and preoperative data of patients classified by surgical approach.

	Anterior	Posterior	Combined	*p* value
Total patients	19	76	58	
Male	8 (42.1%)	52 (68.4%)	40 (69.0%)	0.075
Age (yr)	53.9 ± 9.6	56.3 ± 9.7	55.4 ± 9.0	0.570
Body mass index	24.83 ± 3.71	25.89 ± 3.36	25.22 ± 3.24	0.341
Diabetes	1 (5.3%)	6 (7.9%)	9 (15.5%)	0.366
Hypertension	9 (47.4%)	29 (38.2%)	18 (31.0%)	0.406
Current smoker	1 (5.3%)	18 (23.7%)	14 (24.1%)	0.174
Current drinker	0 (0.0%)	9 (11.8%)	6 (10.3%)	0.375
Symptom duration (mo)	48.8 ± 45.8	55.8 ± 80.3	40.7 ± 69.6	0.334
Follow-up (mo)	18.0 ± 6.6	20.3 ± 8.0	21.6 ± 7.6	0.201
Follow-up ≥ 2 years	3 (15.8%)	28 (36.8%)	26 (44.8%)	0.075
Preoperative JOA score	12.95 ± 1.86	11.73 ± 2.30	11.41 ± 2.50	0.046
ISI on T2WI	7 (36.8%)	44 (57.9%)	45 (77.6%)	0.003
Canal occupancy ratio (%)	37.8 ± 12.5	41.1 ± 14.1	48.0 ± 11.6	0.002
AP cord diameter (mm)	5.00 ± 1.45	3.96 ± 1.56	3.90 ± 1.27	0.013
Ishihara’s curvature index	-3.63 ± 11.19	8.23 ± 11.56	6.98 ± 11.65	< 0.001
Mean Pavlov ratio	0.860 ± 0.105	0.780 ± 0.108	0.741 ± 0.111	< 0.001

JOA indicates Japanese Orthopaedic Association; ISI, increased signal intensity; T2WI, T2-weighted magnetic resonance imaging; AP, anteroposterior. Results are presented as mean ± standard deviation, number (percentage), or number only.

### Comparison of the clinical efficacies of the three surgical approaches

Interestingly, the patients’ preoperative conditions seemed to be most favorable in the anterior group, as indicated by the highest preoperative JOA score (anterior, 12.95 ± 1.86; posterior, 11.73 ± 2.30; combined, 11.41 ± 2.50; *p* = 0.046), the longest anteroposterior cord diameter (anterior, 5.00 ± 1.45 mm; posterior, 3.96 ± 1.56 mm; combined, 3.90 ± 1.27 mm; *p* = 0.013), and the highest Pavlov ratio (anterior, 0.860 ± 0.105; posterior, 0.780 ± 0.108; combined, 0.741 ± 0.111; *p* < 0.001). However, Ishihara’s CI was lowest in the anterior group (anterior, -3.63 ± 11.19; posterior, 8.23 ± 11.56; combined, 6.98 ± 11.65; *p* < 0.001), indicating that the surgeons had preferred the anterior approach for treating cervical kyphosis. There were no significant differences between the posterior and the combined groups in terms of preoperative JOA score, anteroposterior diameter, Pavlov ratio, or Ishihara’s CI. However, among the three groups, the combined group had both the highest incidence of ISI of the spinal cord on T2WI (anterior, 36.8%; posterior, 57.9%; combined, 77.6%; *p* = 0.003) and the highest occupancy ratio of the spinal canal resulting from ventral compression (anterior, 37.8% ± 12.5%; posterior, 41.1% ± 14.1%; combined, 48.0% ± 11.6%; *p* = 0.002).

**Fig 1:**
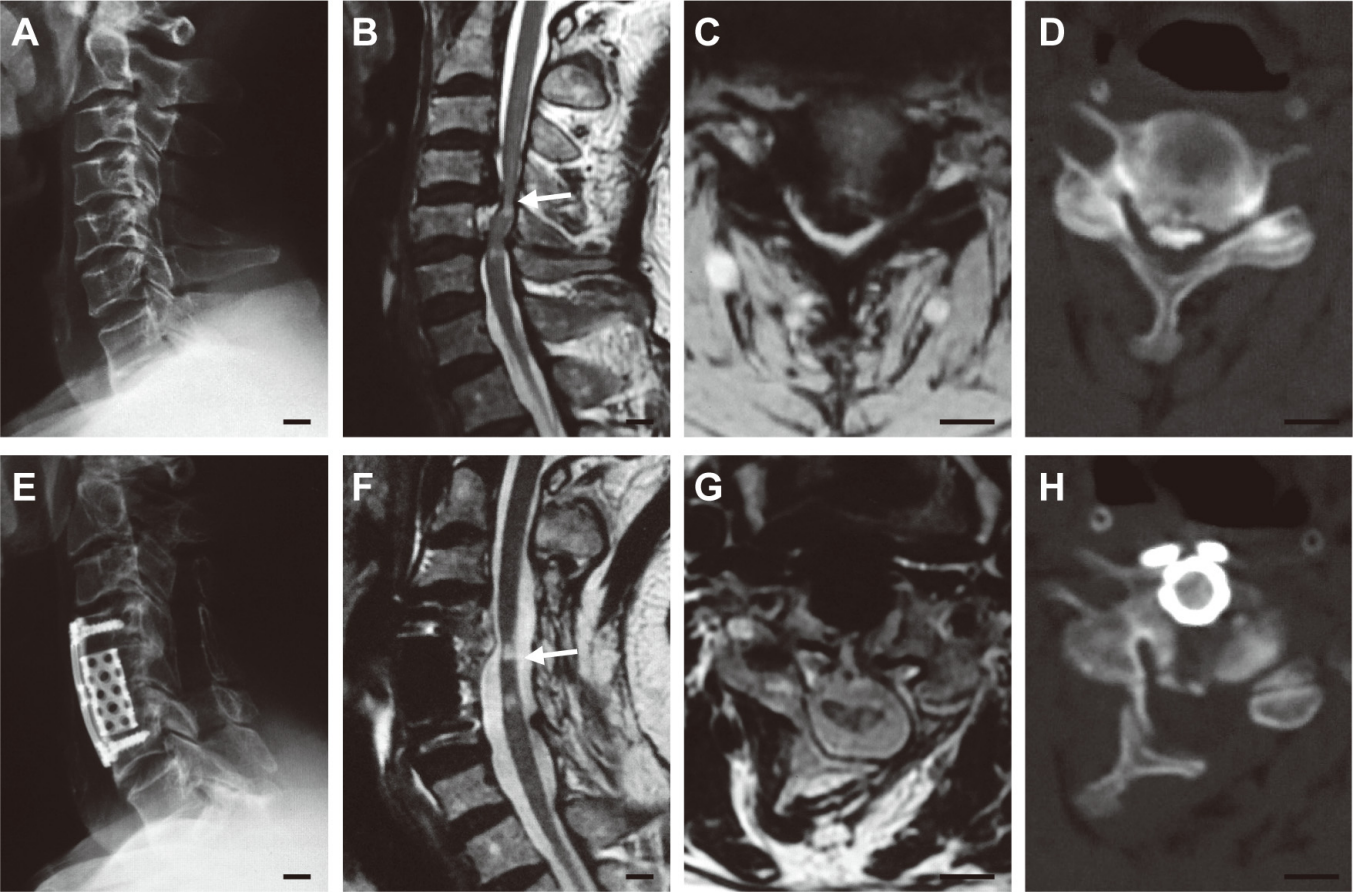
A 53-year-old man who had multilevel cervical spondylotic myelopathy and underwent a combined surgical approach. He suffered from compressions at the C3-6 level (A–D) with a canal occupancy ratio of 54.9% at the maximal compression level (C4-5, compressed by both OPLL and the herniated disc), and was treated with C3-7 laminoplasty followed by C5 ACCF (E–H). Two years after surgery, his JOA score had improved from 8.0 to 16.0 without radiological recompression. (A) Preoperative neutral lateral radiograph. (B) Preoperative T2-weighted sagittal MRI. (C) Preoperative T2-weighted axial MRI at the C4-5 level. (D) Preoperative CT image at the C4-5 level. (E) Postoperative neutral lateral radiograph. (F) Postoperative T2-weighted sagittal MRI. (G) Postoperative T2-weighted axial MRI at the C4-5 level. (H) Postoperative CT image at the C4-5 level. Arrows indicate the maximal compression levels (B, F) where the axial MRI (C, G) and CT (D, H) images should be localized (Scale bar, 10 mm).

All patients in the anterior group had 3 intervertebral segments operated on, while the majority of both the posterior (97.4%) and the combined (96.6%) groups had 4 segments operated on (Table 2). After laminoplasty, 45 (77.6%) and 13 (22.4%) patients in the combined group received 1-level and 2-level anterior decompression, respectively (Fig 1). As a consequence of receiving expanded manipulations, the combined group had the longest operating time (anterior, 104.9 ± 19.4 min; posterior, 104.8 ± 26.2 min; combined, 179.9 ± 46.9 min; *p* < 0.001) and the most blood loss (anterior, 248.4 ± 207.9 ml; posterior, 275.3 ± 140.7 ml; combined, 363.3 ± 256.0 ml; *p* = 0.003) among the three groups. After surgery, patients in both the posterior and the combined groups stayed in the hospital for 5.8 days on average, whereas patients in the anterior group spent an average of 4.2 days in the hospital (*p* < 0.001, Table 2).

**Table 2:** Operation-related parameters of patients classified by surgical approach.

	Anterior	Posterior	Combined	*p* value
Segments operated on*	3.0 ± 0.0	4.0 ± 0.2	4.0 ± 0.2	< 0.001
Anterior segments*				
1	0 (0.0%)	NA	45 (77.6%)	NA
2	0 (0.0%)	NA	13 (22.4%)	NA
3	19 (100.0%)	NA	0 (0.0%)	NA
Posterior segments*				
4	NA	74 (97.4%)	56 (96.6%)	NA
5	NA	2 (2.6%)	2 (3.4%)	NA
Operating Time (min)	104.9 ± 19.4	104.8 ± 26.2	179.9 ± 46.9	< 0.001
Blood Loss (ml)	248.4 ± 207.9	275.3 ± 140.7	363.3 ± 256.0	0.003
Postoperative hospitalized stay (d)	4.2 ± 3.5	5.8 ± 1.7	5.8 ± 2.1	< 0.001

NA indicates not applicable. Results are presented as mean ± standard deviation or number (percentage).

* Segments refer to intervertebral segments; for instance, C3-7 is considered as 4 segments.

At the final follow-up, significant neurological improvements were achieved in all three groups, as assessed by the JOA scores (anterior, from 12.95 ± 1.86 to 15.34 ± 1.40; posterior, from 11.73 ± 2.30 to 14.92 ± 1.56; combined, from 11.41 ± 2.50 to 15.17 ± 1.85; Table 3). Although no significant differences were found among the three groups with respect to postoperative JOA score (*p* = 0.257), JOA improvement was significantly higher in the combined group than in the anterior group (anterior, 2.39 ± 1.40; posterior, 3.19 ± 2.31; combined, 3.77 ± 2.15; *p* = 0.047). Further, the JOA recovery rates in the anterior, posterior, and combined groups were 59.7% ± 25.3%, 54.6% ± 44.9%, and 68.9% ± 23.7%, respectively (*p* = 0.163). Notably, the JOA improvement values and recovery rates seemed to be higher in the combined group than in the posterior group, although the differences were not statistically significant. After adjusting for the demographic and preoperative confounders that are listed in Table 1, no significant differences in neurological outcomes (JOA score, improvement and recovery rate) were observed among the three groups (Table 3).

**Table 3:** Postoperative neurological functions and complications.

	Anterior	Posterior	Combined	*p* value
Postoperative JOA	15.34 ± 1.40	14.92 ± 1.56	15.17 ± 1.85	0.257
JOA improvement	2.39 ± 1.40	3.19 ± 2.31	3.77 ± 2.15	0.047
JOA recover rate (%)	59.7 ± 25.3	54.6 ± 44.9	68.9 ± 23.7	0.163
Adjusted postoperative JOA*	14.81 ± 0.38	14.97 ± 0.17	15.28 ± 0.20	0.424
Adjusted JOA improvement*	3.05 ± 0.38	3.21 ± 0.17	3.52 ± 0.20	0.424
Adjusted JOA recover rate (%)*	57.7 ± 9.2	54.5 ± 4.2	69.6 ± 5.0	0.080
Complications				
Dysphagia	1 (5.3%)	0 (0.0%)	3 (5.2%)	0.087
Transient dysphonia	5 (26.3%)	0 (0.0%)	4 (6.9%)	< 0.001
Transient nerve root paresthesia	2 (10.5%)	8 (10.5%)	1 (1.7%)	0.096
Wound complications	0 (0.0%)	3 (3.9%)	1 (1.7%)	0.785
CSF leakage	0 (0.0%)	0 (0.0%)	2 (3.4%)	0.376
Axial pain	3 (15.8%)	13 (17.1%)	6 (10.3%)	0.555
Axial symptoms	7 (36.8%)	29 (38.2%)	21 (36.2%)	0.972
C5 palsy	0 (0.0%)	3 (3.9%)	1 (1.7%)	0.785
Reoperation	0 (0.0%)	3 (3.9%)	0 (0.0%)	0.360
Complication rate	11 (57.9%)	38 (50.0%)	26 (44.8%)	0.600

JOA indicates Japanese Orthopaedic Association; CSF, cerebrospinal fluid. The unadjusted results are presented as mean ± standard deviation or number (percentage), while the adjusted results (indicated by *) are presented as mean ± standard error.

* Adjusted for the demographic and preoperative parameters, including sex, age, body mass index, diabetes, hypertension, smoking, drinking, duration of symptoms, preoperative JOA score, increased signal intensity on T2-weighted MR imaging, canal occupancy ratio, anteroposterior diameter, Pavlov ratio, and Ishihara’s curvature index.

The overall complication rates of the three groups did not differ significantly from each other (*p* = 0.600, Table 3). Five subjects (26.3%) in the anterior group and 4 subjects (6.9%) in the combined group suffered from temporary dysphonia after surgery, but no one in the posterior group experienced this complication (*p* < 0.001). Similarly, dysphagia was only reported in the anterior (1 subject, 5.3%) and combined (3 subjects, 5.2%) groups. In contrast, all 3 subjects who underwent reoperation were in the posterior group (3.9%). Two of these subjects received a reoperation for lamina closure, and one of these patients received a reoperation for a planned two-stage operation. There were also 2 cerebrospinal fluid (CSF) leakages (3.4%), 1 superficial wound infection (1.7%), 1 transient C5 palsy (1.7%), 21 axial symptoms (36.2%), and 6 axial pains (10.3%) that occurred in the combined group. With the exception of transient dysphonia, however, there were no statistically significant differences between the rates of the complications in the three surgical groups.

### Is an occupancy ratio-related indication appropriate for the combined approach?

The anterior-only, posterior-only, and combined approaches were found to have equivalent clinical efficacy, which raises the question, “What surgical indications are appropriate for the combined approach?” In our clinical practice, we arbitrarily choose the combined approach for multilevel cervical spondylosis associated with severe ventral compression, especially when the occupancy ratio of the spinal canal exceeded 50% [14, 25]. However, few studies have investigated the appropriateness of this occupancy ratio-related indication in clinical practice.

For this purpose, further analyses were conducted in the subgroups of patients with occupancy ratios of at least 50% (Table 4). The anterior subgroup was excluded from these analyses because of the limited sample size of patients with occupancy ratios ≥ 50% (number of subjects: anterior, 3; posterior, 20; combined, 24), and the remaining two groups were found to have similar occupancy ratios at baseline (posterior, 60.0% ± 6.7%; combined, 58.8% ± 6.7%; *p* = 0.560) and preoperative JOA scores (posterior, 11.13 ± 2.44; combined, 11.06 ± 2.41; *p* = 0.933). The posterior and combined approaches did not differ significantly in terms of JOA-related clinical outcomes at the final follow-up (preoperative JOA score, *p* = 0.598; JOA improvement, *p* = 0.782; JOA recover rate, *p* = 0.838; Table 4).

**Table 4:** Subgroup analyses of the MCSM patients with occupancy ratios ≥ 50%.

Occupancy ratio ≥ 50%	Anterior*	Posterior	Combined	*p* value*
Patient number	3	20	24	
Occupancy ratio (%)	59.2 ± 2.5	60.0 ± 6.7	58.8 ± 6.7	0.560
Preoperative JOA	11.00 ± 0.87	11.13 ± 2.44	11.06 ± 2.41	0.933
Postoperative JOA	13.83 ± 1.15	15.28 ± 1.15	15.00 ± 2.06	0.598
JOA improvement	2.83 ± 0.29	4.15 ± 2.67	3.94 ± 2.39	0.782
JOA recover rate (%)	48.3 ± 10.9	64.3 ± 42.5	66.5 ± 26.7	0.838

JOA indicates Japanese Orthopaedic Association. Results are presented as mean ± standard deviation or number only.

* Because of the limited sample size (n = 3), the anterior approach was excluded from the statistical comparisons.

**Fig 2:**
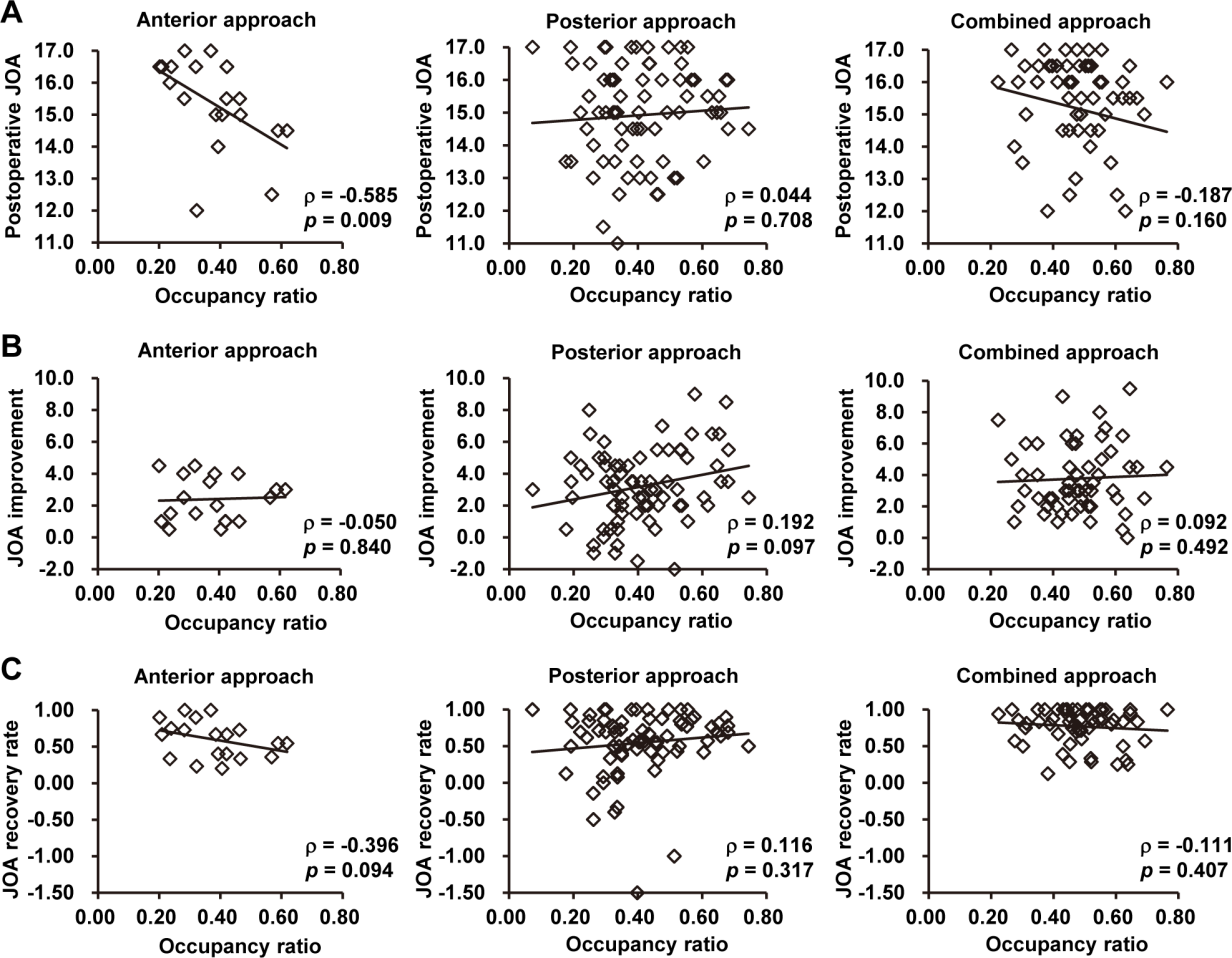
Scatterplots of the relationships between the occupancy ratio and neurological outcomes. (A) Postoperative JOA score versus occupancy ratio. (B) JOA improvement versus occupancy ratio. (C) JOA recovery rate versus occupancy ratio. Data were grouped by surgical approach. Each graph presents Spearman’s rank-correlation coefficient (ρ), the associated *p*-value, and a best-fit line.

To further clarify the role of the occupancy ratio in surgical decision-making for MCSM, the relationship between the occupancy ratio and postoperative neurological recovery was investigated using correlation analysis. First, scatterplots were created of all the patients’ data, as grouped by surgical approach, with the occupancy ratio on the x-axis and the postoperative JOA score, improvement, or recovery rate on the y-axis (Fig 2). Next, Spearman’s rank correlation test was performed to determine the associations between these variables. Except for a moderately negative correlation between the occupancy ratio and postoperative JOA in the anterior group (Spearman’s ρ = -0.585, *p* = 0.009), no other correlations were statistically significant, suggesting that occupancy ratio does not have a negative association with the surgical efficacy of the posterior or combined approach. Since the sample size of the anterior group was relatively small (n = 19) and the postoperative JOA score was also affected by the preoperative JOA (Spearman’s ρ = -0.650, *p* = 0.003), the occupancy ratio’s role in the efficacy of the anterior surgical approach was inconclusive.

## Discussion

Although increasingly many studies have assessed the efficacy of a combined approach in the treatment of MCSM, a clear consensus has not been reached. Konya *et al* [6] and Karpova *et al* [22] suggested that the combined approach was as effective as other approaches for symptom relief. The first part of our analysis also demonstrated that all three surgical approaches could effectively improve the neurological function of MCSM patients, if applied appropriately. Further, the anterior, posterior, and combined approaches were observed to have equivalent functional outcomes. Interestingly, the combined approach achieved much better neurological improvements than did the anterior approach, but the differences were eliminated after adjusting for baseline confounders. This result is similar to that of a recent multicenter study by Fehlings *et al* [13], in which patients chosen for the anterior approach usually had less severe conditions, as assessed by their preoperative JOA scores. It is possible that because the preoperative conditions were less severe, there was relatively limited room for improvement in neurological function. In another retrospective study, Wen *et al* [7] claimed that the combined approach consistently had the highest JOA recovery rate, both before and after multivariable adjustment. In the present study, the combined group had a higher JOA recovery rate than the other two groups, but the difference did not appear to be statistically significant. This discrepancy may result from variation among the research subjects and the surgeons’ familiarity with different techniques.

In addition to efficacy, safety is another important factor that influences the application of the combined approach in clinical practice. After prospectively investigating 302 patients from the AOSpine North America Cervical Spondylotic Myelopathy Study, Fehlings *et al* [19] claimed that the combined approach was associated with increased rates of postoperative complications, especially dysphagia. Sembrano *et al* [26] have reported that, as compared with the anterior-only approach, the combined anterior-posterior approach could significantly reduce the incidence of reoperations caused by pseudarthrosis, but could not reduce the overall reoperation rate. In the present study, we found that the rates of postoperative complications, including dysphagia and reoperation, were all comparable among the three approaches, with the exception of a higher incidence of transient dysphonia in the anterior group. In fact, none of the 58 patients in the combined group underwent a reoperation, while 3 patients in the posterior group had reoperations. Thus, our results suggest that the combined approach is at least as safe as the other approaches, although it usually results in the most surgical trauma (as indicated by increased operating times and blood loss).

The surgical indication for the combined approach in CSM treatment remains controversial. Because of its disadvantages, such as technical complexity, prolonged surgical times, and increased blood loss, the combined approach used to be a surgical strategy that was reserved for complicated cases of CSM [8, 27]. Generally speaking, patients who have CSM with severe kyphotic deformity, osteoporosis, or instability should be considered for the combined approach [15]. MCSM associated with severe ventral compression is another important indication for the addition of anterior decompression after posterior manipulations, especially when the occupancy ratio of the spinal canal exceeds 50%, [14, 25]. However, there has been little evidence in the literature to verify this occupancy ratio-related indication. In the second part of this study, we focused on the subgroup of patients with high occupancy ratios to examine whether the combined approach was suitable for treating MCSM with large ventral compressions. We performed a correlation analysis to test whether increases in the occupancy ratio were correlated with compromised outcomes when using the anterior-only or posterior-only approach for MCSM. To our surprise, both results implied that the occupancy ratio of the spinal canal at the maximal compression level might not be an appropriate indication for the combined approach. Further studies are required to determine the most suitable indication for the combined approach.

The major limitations to our study are that it had a retrospective design and lacked randomization, making biases and confounding difficult to control. Further, although the small size of the anterior group is expected and reasonable, given that surgeons usually prefer the posterior and combined approaches for MSCM treatment [8], this small number of patients presented a limitation to our additional analyses. Our study was also limited by its reliance on data from the final follow-up; if we had analyzed multiple, periodic follow-ups, it would have been possible to investigate the dynamics of the recovery processes after surgery. Additionally, all of our results were limited to selected patients with normal sagittal alignment or mild degrees of kyphosis. Finally, it is possible that the complication rates were lower than those of other study cohorts because of the retrospective nature of our investigation.

## Conclusions

This retrospective study demonstrates that the anterior, posterior, and combined surgical strategies are all effective and reliable options for the treatment of MCSM. After adjustment for potential confounders, patients treated with the anterior, posterior, and one-stage combined posterior-anterior approaches experience equivalent neurological recoveries when the current surgical indications are employed. Among patients with heavy ventral compression, the neurological recoveries following treatment with the combined approach do not differ significantly from those following treatment with the posterior approach. Furthermore, there is no significant association between high occupancy ratios and compromised recovery in the MCSM patients treated with posterior-only approach, indicating that there are important limitations to the occupancy ratio-related surgical indication for the combined approach. Further studies are required in the future, especially prospective, randomized, multicenter controlled trials with long-term follow-up.
